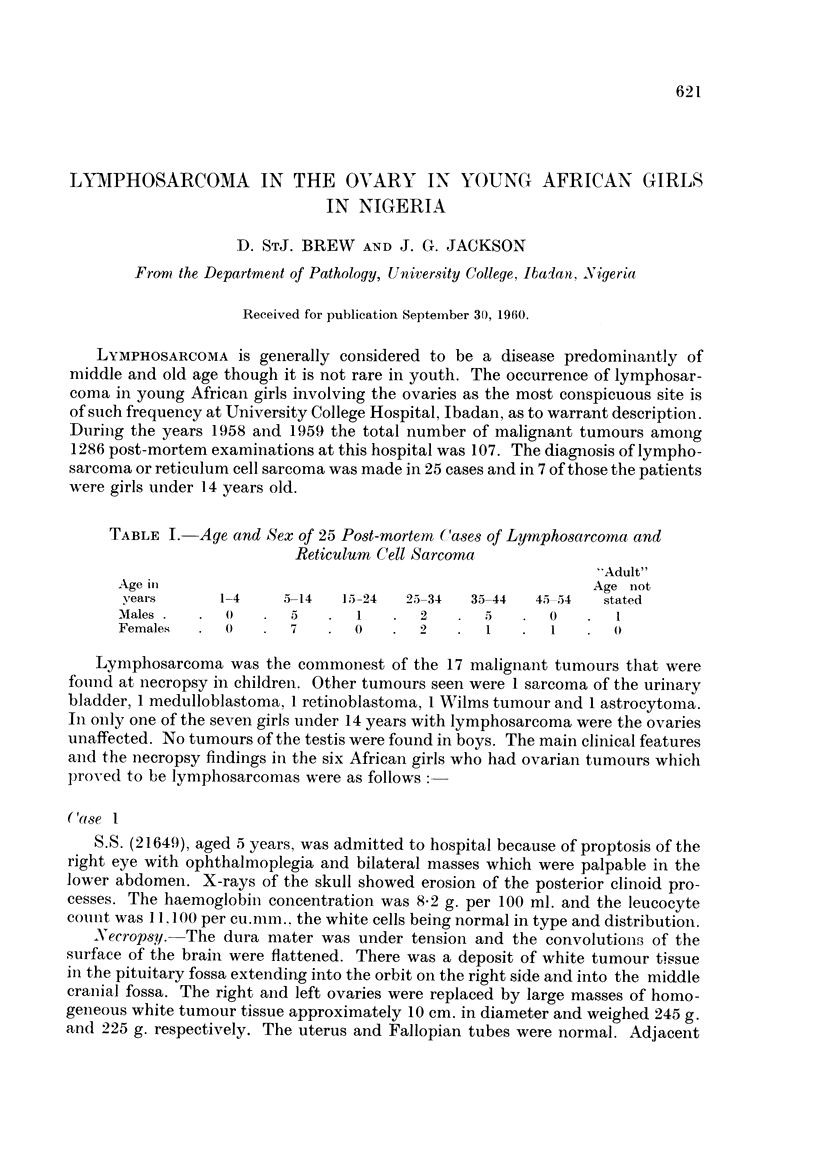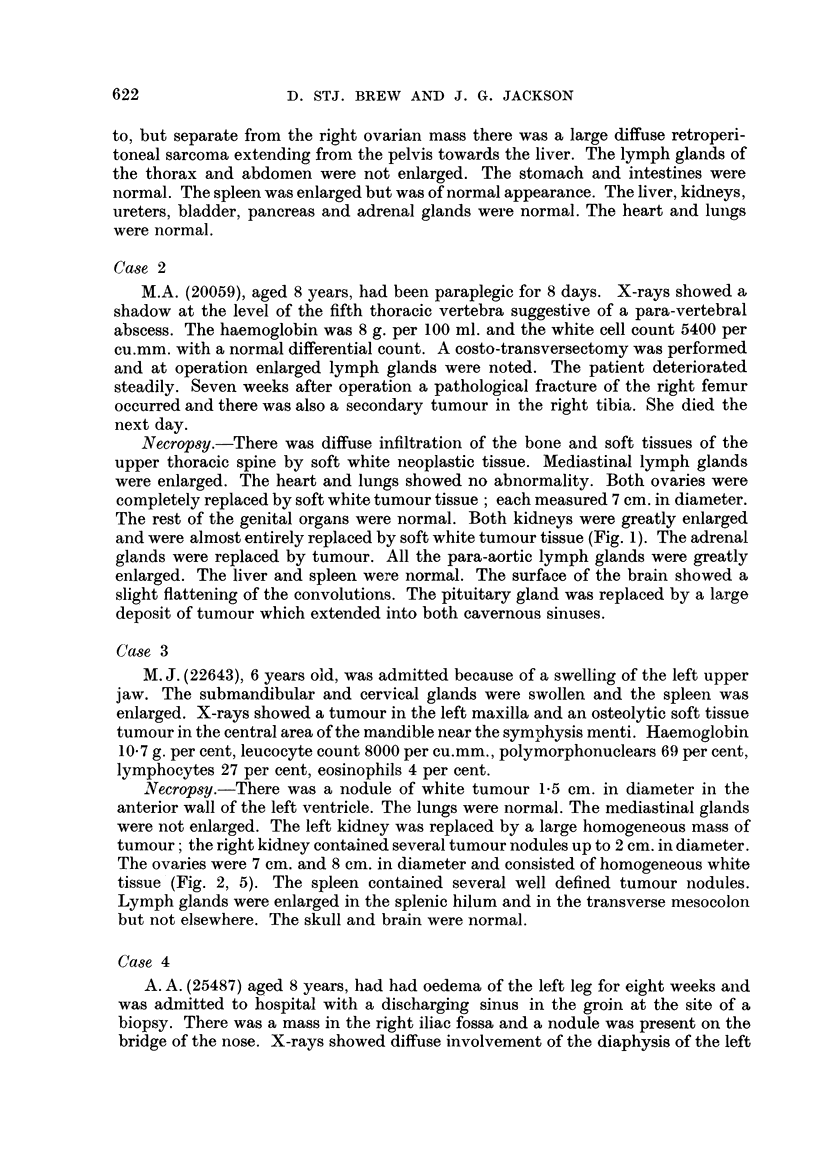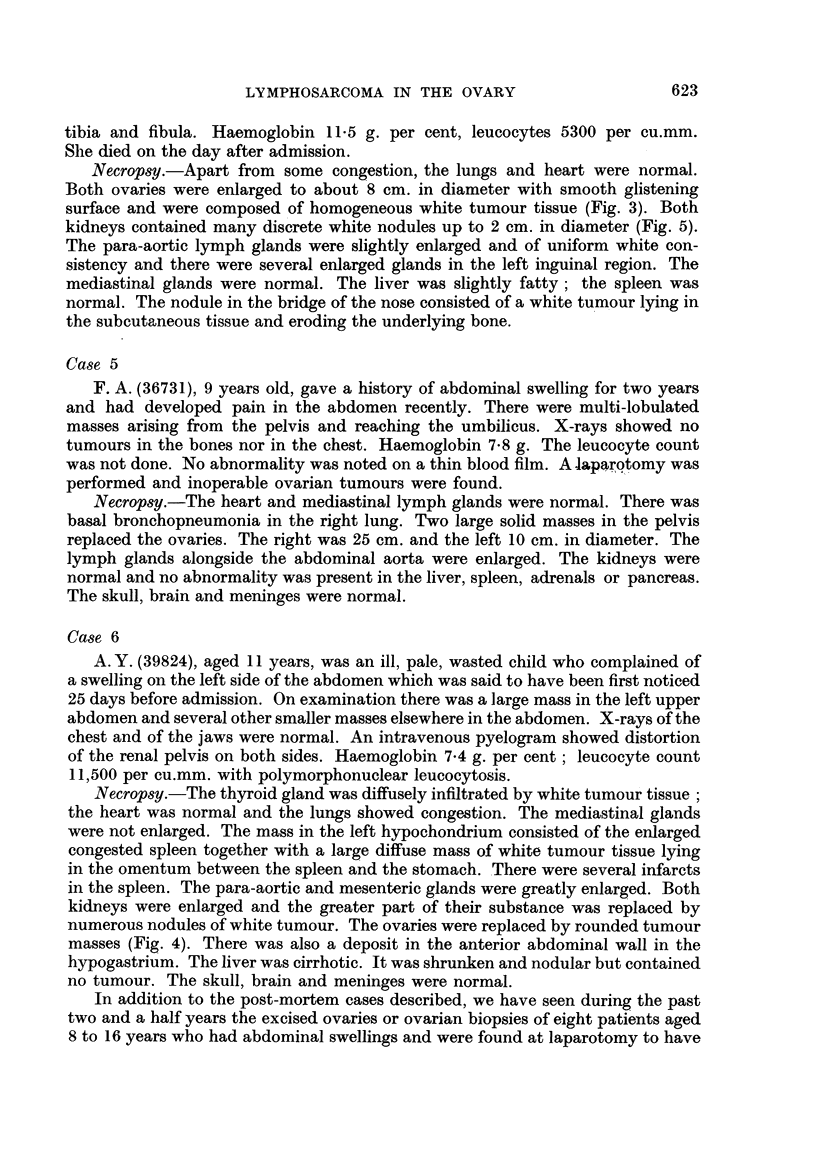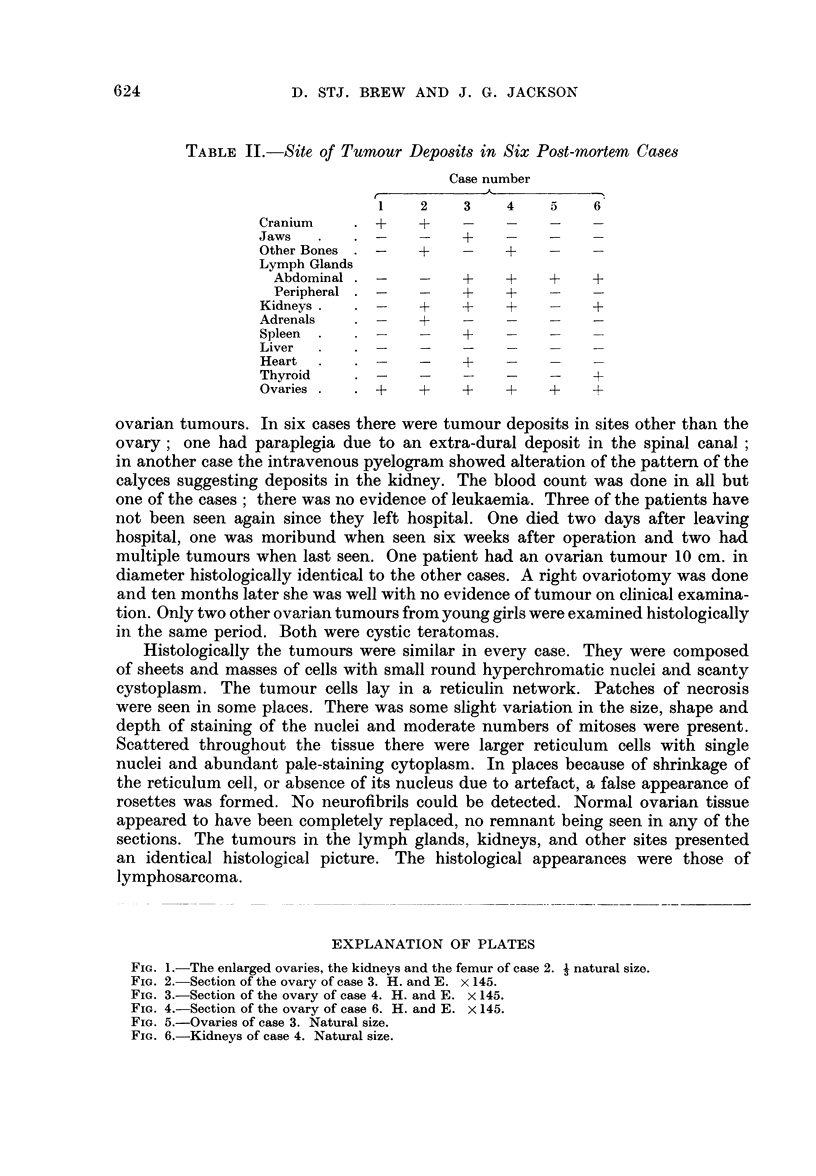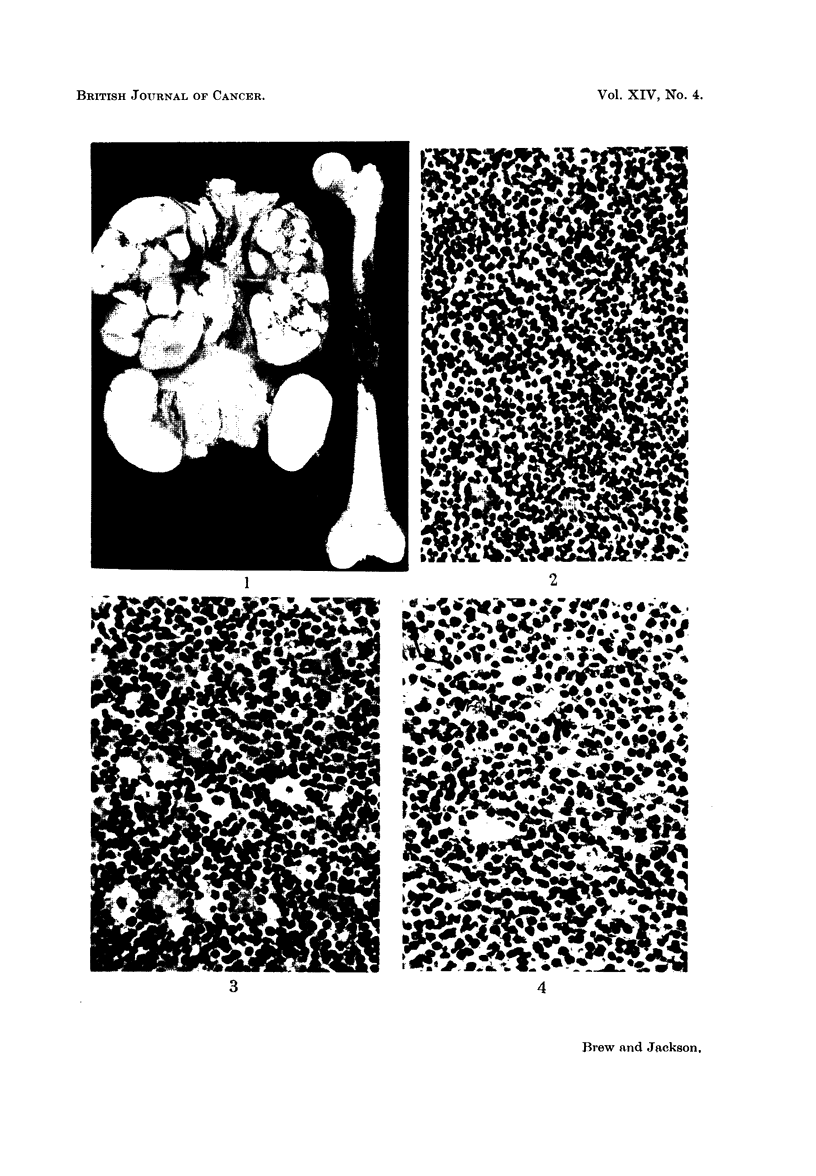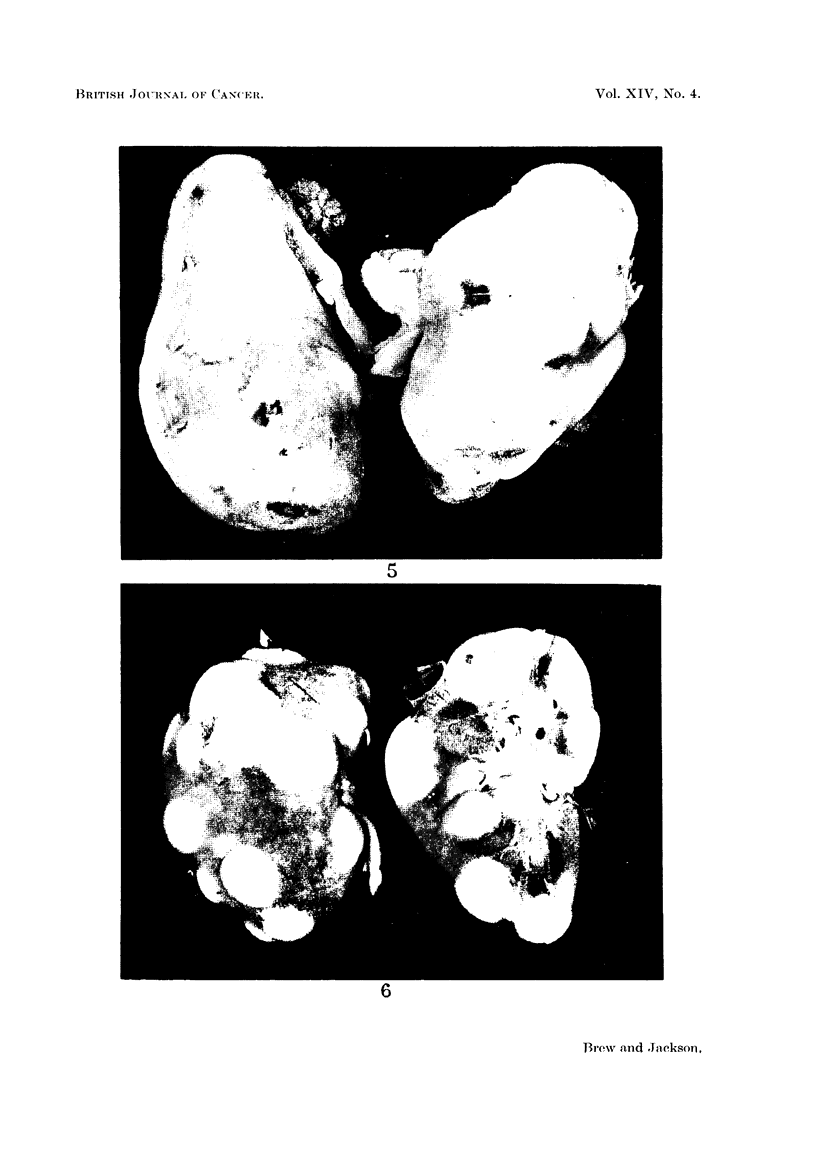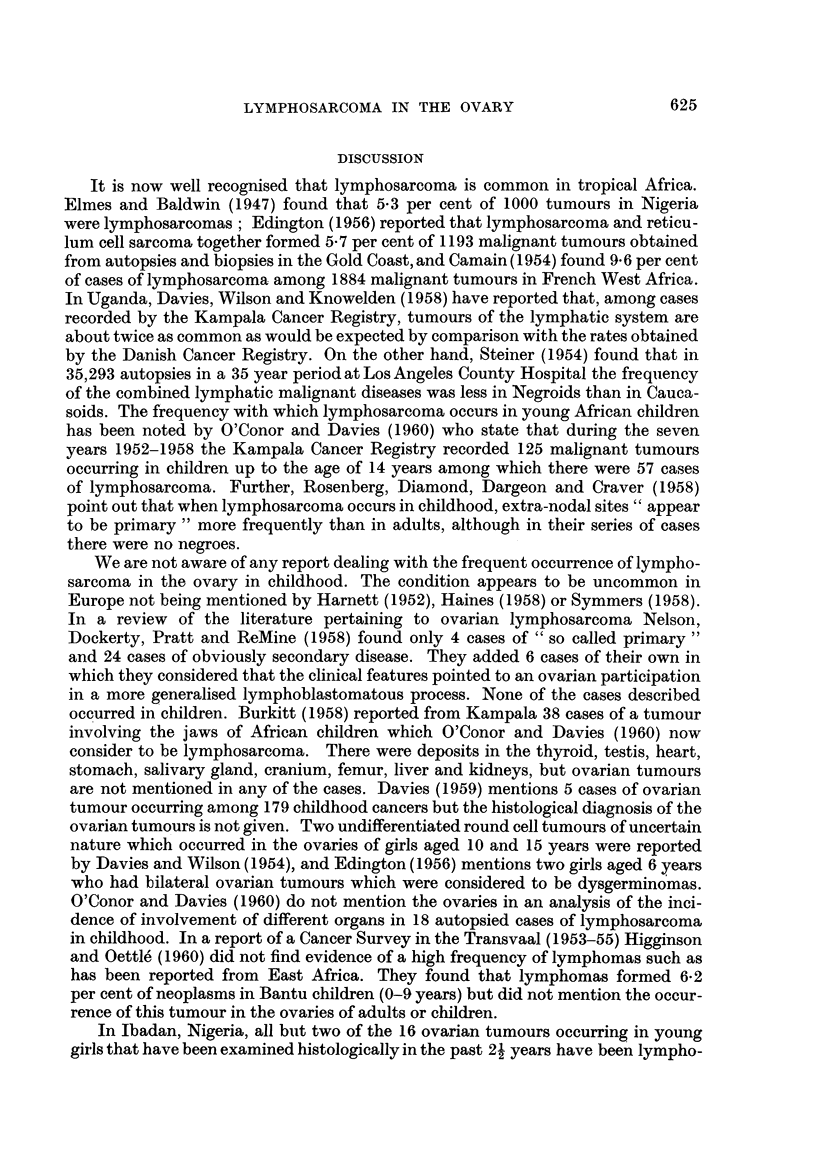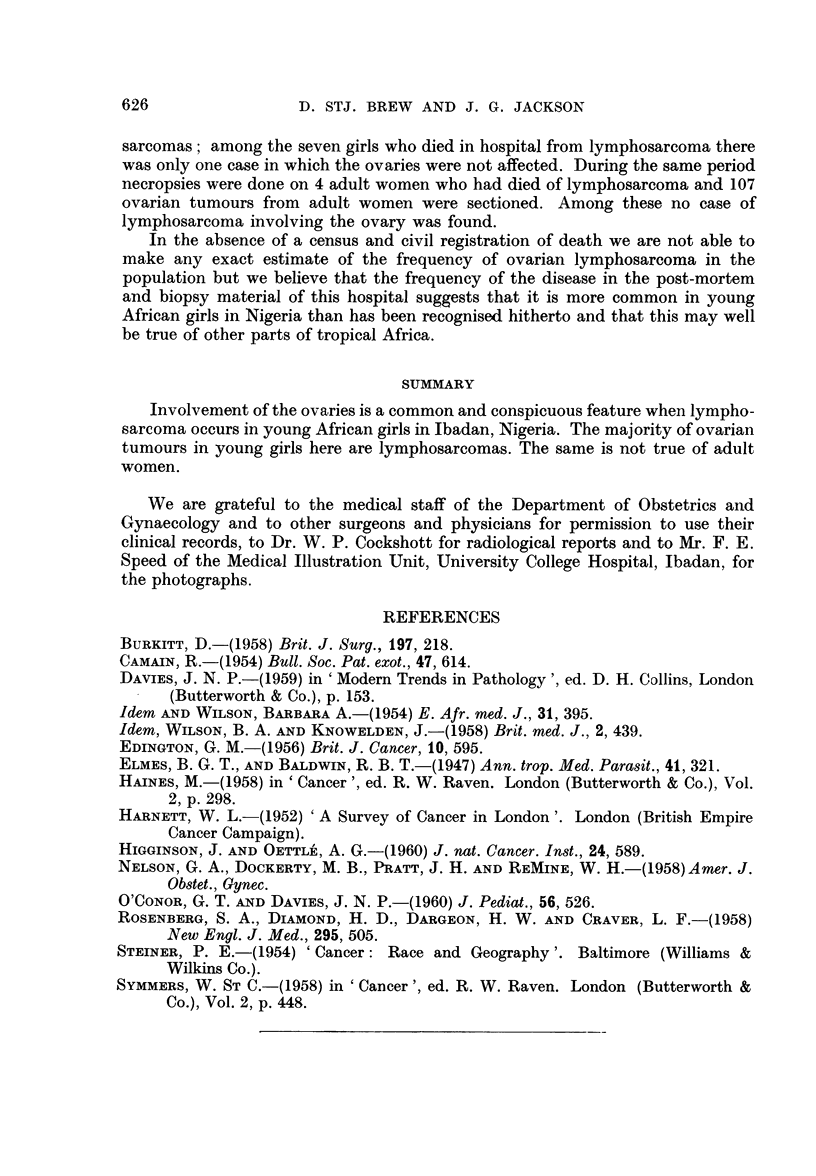# Lymphosarcoma in the Ovary in Young African Girls in Nigeria

**DOI:** 10.1038/bjc.1960.68

**Published:** 1960-12

**Authors:** D. StJ. Brew, J. G. Jackson

## Abstract

**Images:**


### LYMPHOSARCOMA IN THE OVARY IN YOUNG AFRICAN GIRLS IN NIGERIA

D. StJ. BREW AND J. G. JACKSON

*From the Department of Pathology, University College, Ibadan, Nigeria*

Received for publication September 30, 1960.

LYMPHOSARCOMA is generally considered to be a disease predominantly of middle and old age though it is not rare in youth. The occurrence of lymphosarcoma in young African girls involving the ovaries as the most conspicuous site is of such frequency at University College Hospital, Ibadan, as to warrant description. During the years 1958 and 1959 the total number of malignant tumours among 1286 post-mortem examinations at this hospital was 107. The diagnosis of lymphosarcoma or reticulum cell sarcoma was made in 25 cases and in 7 of those the patients were girls under 14 years old.

TABLE I.—*Age and Sex of 25 Post-mortem Cases of Lymphosarcoma and Reticulum Cell Sarcoma*

| Age in years | 1–4 | 5–14 | 15–24 | 25–34 | 35–44 | 45–54 | "Adult" Age not stated |
|---|---|---|---|---|---|---|---|
| Males | 0 | 5 | 1 | 2 | 5 | 0 | 1 |
| Females | 0 | 7 | 0 | 2 | 1 | 1 | 0 |

Lymphosarcoma was the commonest of the 17 malignant tumours that were found at necropsy in children. Other tumours seen were 1 sarcoma of the urinary bladder, 1 medulloblastoma, 1 retinoblastoma, 1 Wilms tumour and 1 astrocytoma. In only one of the seven girls under 14 years with lymphosarcoma were the ovaries unaffected. No tumours of the testis were found in boys. The main clinical features and the necropsy findings in the six African girls who had ovarian tumours which proved to be lymphosarcomas were as follows :—

*Case* 1

S.S. (21649), aged 5 years, was admitted to hospital because of proptosis of the right eye with ophthalmoplegia and bilateral masses which were palpable in the lower abdomen. X-rays of the skull showed erosion of the posterior clinoid processes. The haemoglobin concentration was 8·2 g. per 100 ml. and the leucocyte count was 11,100 per cu.mm., the white cells being normal in type and distribution.

*Necropsy.*—The dura mater was under tension and the convolutions of the surface of the brain were flattened. There was a deposit of white tumour tissue in the pituitary fossa extending into the orbit on the right side and into the middle cranial fossa. The right and left ovaries were replaced by large masses of homogeneous white tumour tissue approximately 10 cm. in diameter and weighed 245 g. and 225 g. respectively. The uterus and Fallopian tubes were normal. Adjacent to, but separate from the right ovarian mass there was a large diffuse retroperitoneal sarcoma extending from the pelvis towards the liver. The lymph glands of the thorax and abdomen were not enlarged. The stomach and intestines were normal. The spleen was enlarged but was of normal appearance. The liver, kidneys, ureters, bladder, pancreas and adrenal glands were normal. The heart and lungs were normal.

*Case* 2

M.A. (20059), aged 8 years, had been paraplegic for 8 days. X-rays showed a shadow at the level of the fifth thoracic vertebra suggestive of a para-vertebral abscess. The haemoglobin was 8 g. per 100 ml. and the white cell count 5400 per cu.mm. with a normal differential count. A costo-transversectomy was performed and at operation enlarged lymph glands were noted. The patient deteriorated steadily. Seven weeks after operation a pathological fracture of the right femur occurred and there was also a secondary tumour in the right tibia. She died the next day.

*Necropsy*.—There was diffuse infiltration of the bone and soft tissues of the upper thoracic spine by soft white neoplastic tissue. Mediastinal lymph glands were enlarged. The heart and lungs showed no abnormality. Both ovaries were completely replaced by soft white tumour tissue ; each measured 7 cm. in diameter. The rest of the genital organs were normal. Both kidneys were greatly enlarged and were almost entirely replaced by soft white tumour tissue (Fig. 1). The adrenal glands were replaced by tumour. All the para-aortic lymph glands were greatly enlarged. The liver and spleen were normal. The surface of the brain showed a slight flattening of the convolutions. The pituitary gland was replaced by a large deposit of tumour which extended into both cavernous sinuses.

*Case* 3

M. J. (22643), 6 years old, was admitted because of a swelling of the left upper jaw. The submandibular and cervical glands were swollen and the spleen was enlarged. X-rays showed a tumour in the left maxilla and an osteolytic soft tissue tumour in the central area of the mandible near the symphysis menti. Haemoglobin 10·7 g. per cent, leucocyte count 8000 per cu.mm., polymorphonuclears 69 per cent, lymphocytes 27 per cent, eosinophils 4 per cent.

*Necropsy*.—There was a nodule of white tumour 1·5 cm. in diameter in the anterior wall of the left ventricle. The lungs were normal. The mediastinal glands were not enlarged. The left kidney was replaced by a large homogeneous mass of tumour ; the right kidney contained several tumour nodules up to 2 cm. in diameter. The ovaries were 7 cm. and 8 cm. in diameter and consisted of homogeneous white tissue (Fig. 2, 5). The spleen contained several well defined tumour nodules. Lymph glands were enlarged in the splenic hilum and in the transverse mesocolon but not elsewhere. The skull and brain were normal.

*Case* 4

A. A. (25487) aged 8 years, had had oedema of the left leg for eight weeks and was admitted to hospital with a discharging sinus in the groin at the site of a biopsy. There was a mass in the right iliac fossa and a nodule was present on the bridge of the nose. X-rays showed diffuse involvement of the diaphysis of the left tibia and fibula. Haemoglobin 11·5 g. per cent, leucocytes 5300 per cu.mm. She died on the day after admission.

*Necropsy.*—Apart from some congestion, the lungs and heart were normal. Both ovaries were enlarged to about 8 cm. in diameter with smooth glistening surface and were composed of homogeneous white tumour tissue (Fig. 3). Both kidneys contained many discrete white nodules up to 2 cm. in diameter (Fig. 5). The para-aortic lymph glands were slightly enlarged and of uniform white consistency and there were several enlarged glands in the left inguinal region. The mediastinal glands were normal. The liver was slightly fatty ; the spleen was normal. The nodule in the bridge of the nose consisted of a white tumour lying in the subcutaneous tissue and eroding the underlying bone.

*Case* 5

F. A. (36731), 9 years old, gave a history of abdominal swelling for two years and had developed pain in the abdomen recently. There were multi-lobulated masses arising from the pelvis and reaching the umbilicus. X-rays showed no tumours in the bones nor in the chest. Haemoglobin 7·8 g. The leucocyte count was not done. No abnormality was noted on a thin blood film. A laparotomy was performed and inoperable ovarian tumours were found.

*Necropsy.*—The heart and mediastinal lymph glands were normal. There was basal bronchopneumonia in the right lung. Two large solid masses in the pelvis replaced the ovaries. The right was 25 cm. and the left 10 cm. in diameter. The lymph glands alongside the abdominal aorta were enlarged. The kidneys were normal and no abnormality was present in the liver, spleen, adrenals or pancreas. The skull, brain and meninges were normal.

*Case* 6

A. Y. (39824), aged 11 years, was an ill, pale, wasted child who complained of a swelling on the left side of the abdomen which was said to have been first noticed 25 days before admission. On examination there was a large mass in the left upper abdomen and several other smaller masses elsewhere in the abdomen. X-rays of the chest and of the jaws were normal. An intravenous pyelogram showed distortion of the renal pelvis on both sides. Haemoglobin 7·4 g. per cent ; leucocyte count 11,500 per cu.mm. with polymorphonuclear leucocytosis.

*Necropsy.*—The thyroid gland was diffusely infiltrated by white tumour tissue ; the heart was normal and the lungs showed congestion. The mediastinal glands were not enlarged. The mass in the left hypochondrium consisted of the enlarged congested spleen together with a large diffuse mass of white tumour tissue lying in the omentum between the spleen and the stomach. There were several infarcts in the spleen. The para-aortic and mesenteric glands were greatly enlarged. Both kidneys were enlarged and the greater part of their substance was replaced by numerous nodules of white tumour. The ovaries were replaced by rounded tumour masses (Fig. 4). There was also a deposit in the anterior abdominal wall in the hypogastrium. The liver was cirrhotic. It was shrunken and nodular but contained no tumour. The skull, brain and meninges were normal.

In addition to the post-mortem cases described, we have seen during the past two and a half years the excised ovaries or ovarian biopsies of eight patients aged 8 to 16 years who had abdominal swellings and were found at laparotomy to have

TABLE II.—*Site of Tumour Deposits in Six Post-mortem Cases*

| | Case number | | | | | |
|---|---|---|---|---|---|---|
| | 1 | 2 | 3 | 4 | 5 | 6 |
| Cranium . | + | + | – | – | – | – |
| Jaws . . | – | – | + | – | – | – |
| Other Bones . | – | + | – | + | – | – |
| Lymph Glands | | | | | | |
| Abdominal . | – | – | + | + | + | + |
| Peripheral . | – | – | + | + | – | – |
| Kidneys . . | – | + | + | + | – | + |
| Adrenals . | – | + | – | – | – | – |
| Spleen . . | – | – | + | – | – | – |
| Liver . . | – | – | – | – | – | – |
| Heart . . | – | – | + | – | – | – |
| Thyroid . | – | – | – | – | – | + |
| Ovaries . . | + | + | + | + | + | + |

ovarian tumours. In six cases there were tumour deposits in sites other than the ovary ; one had paraplegia due to an extra-dural deposit in the spinal canal ; in another case the intravenous pyelogram showed alteration of the pattern of the calyces suggesting deposits in the kidney. The blood count was done in all but one of the cases ; there was no evidence of leukaemia. Three of the patients have not been seen again since they left hospital. One died two days after leaving hospital, one was moribund when seen six weeks after operation and two had multiple tumours when last seen. One patient had an ovarian tumour 10 cm. in diameter histologically identical to the other cases. A right ovariotomy was done and ten months later she was well with no evidence of tumour on clinical examination. Only two other ovarian tumours from young girls were examined histologically in the same period. Both were cystic teratomas.

Histologically the tumours were similar in every case. They were composed of sheets and masses of cells with small round hyperchromatic nuclei and scanty cystoplasm. The tumour cells lay in a reticulin network. Patches of necrosis were seen in some places. There was some slight variation in the size, shape and depth of staining of the nuclei and moderate numbers of mitoses were present. Scattered throughout the tissue there were larger reticulum cells with single nuclei and abundant pale-staining cytoplasm. In places because of shrinkage of the reticulum cell, or absence of its nucleus due to artefact, a false appearance of rosettes was formed. No neurofibrils could be detected. Normal ovarian tissue appeared to have been completely replaced, no remnant being seen in any of the sections. The tumours in the lymph glands, kidneys, and other sites presented an identical histological picture. The histological appearances were those of lymphosarcoma.

---

EXPLANATION OF PLATES

FIG. 1.—The enlarged ovaries, the kidneys and the femur of case 2. $\frac{1}{3}$ natural size.
FIG. 2.—Section of the ovary of case 3. H. and E. $\times 145$.
FIG. 3.—Section of the ovary of case 4. H. and E. $\times 145$.
FIG. 4.—Section of the ovary of case 6. H. and E. $\times 145$.
FIG. 5.—Ovaries of case 3. Natural size.
FIG. 6.—Kidneys of case 4. Natural size.

Brew and Jackson.

5

6

### DISCUSSION

It is now well recognised that lymphosarcoma is common in tropical Africa. Elmes and Baldwin (1947) found that 5·3 per cent of 1000 tumours in Nigeria were lymphosarcomas ; Edington (1956) reported that lymphosarcoma and reticulum cell sarcoma together formed 5·7 per cent of 1193 malignant tumours obtained from autopsies and biopsies in the Gold Coast, and Camain (1954) found 9·6 per cent of cases of lymphosarcoma among 1884 malignant tumours in French West Africa. In Uganda, Davies, Wilson and Knowelden (1958) have reported that, among cases recorded by the Kampala Cancer Registry, tumours of the lymphatic system are about twice as common as would be expected by comparison with the rates obtained by the Danish Cancer Registry. On the other hand, Steiner (1954) found that in 35,293 autopsies in a 35 year period at Los Angeles County Hospital the frequency of the combined lymphatic malignant diseases was less in Negroids than in Caucasoids. The frequency with which lymphosarcoma occurs in young African children has been noted by O'Conor and Davies (1960) who state that during the seven years 1952–1958 the Kampala Cancer Registry recorded 125 malignant tumours occurring in children up to the age of 14 years among which there were 57 cases of lymphosarcoma. Further, Rosenberg, Diamond, Dargeon and Craver (1958) point out that when lymphosarcoma occurs in childhood, extra-nodal sites " appear to be primary " more frequently than in adults, although in their series of cases there were no negroes.

We are not aware of any report dealing with the frequent occurrence of lymphosarcoma in the ovary in childhood. The condition appears to be uncommon in Europe not being mentioned by Harnett (1952), Haines (1958) or Symmers (1958). In a review of the literature pertaining to ovarian lymphosarcoma Nelson, Dockerty, Pratt and ReMine (1958) found only 4 cases of " so called primary " and 24 cases of obviously secondary disease. They added 6 cases of their own in which they considered that the clinical features pointed to an ovarian participation in a more generalised lymphoblastomatous process. None of the cases described occurred in children. Burkitt (1958) reported from Kampala 38 cases of a tumour involving the jaws of African children which O'Conor and Davies (1960) now consider to be lymphosarcoma. There were deposits in the thyroid, testis, heart, stomach, salivary gland, cranium, femur, liver and kidneys, but ovarian tumours are not mentioned in any of the cases. Davies (1959) mentions 5 cases of ovarian tumour occurring among 179 childhood cancers but the histological diagnosis of the ovarian tumours is not given. Two undifferentiated round cell tumours of uncertain nature which occurred in the ovaries of girls aged 10 and 15 years were reported by Davies and Wilson (1954), and Edington (1956) mentions two girls aged 6 years who had bilateral ovarian tumours which were considered to be dysgerminomas. O'Conor and Davies (1960) do not mention the ovaries in an analysis of the incidence of involvement of different organs in 18 autopsied cases of lymphosarcoma in childhood. In a report of a Cancer Survey in the Transvaal (1953–55) Higginson and Oettlé (1960) did not find evidence of a high frequency of lymphomas such as has been reported from East Africa. They found that lymphomas formed 6·2 per cent of neoplasms in Bantu children (0–9 years) but did not mention the occurrence of this tumour in the ovaries of adults or children.

In Ibadan, Nigeria, all but two of the 16 ovarian tumours occurring in young girls that have been examined histologically in the past 2½ years have been lymphosarcomas ; among the seven girls who died in hospital from lymphosarcoma there was only one case in which the ovaries were not affected. During the same period necropsies were done on 4 adult women who had died of lymphosarcoma and 107 ovarian tumours from adult women were sectioned. Among these no case of lymphosarcoma involving the ovary was found.

In the absence of a census and civil registration of death we are not able to make any exact estimate of the frequency of ovarian lymphosarcoma in the population but we believe that the frequency of the disease in the post-mortem and biopsy material of this hospital suggests that it is more common in young African girls in Nigeria than has been recognised hitherto and that this may well be true of other parts of tropical Africa.

### SUMMARY

Involvement of the ovaries is a common and conspicuous feature when lymphosarcoma occurs in young African girls in Ibadan, Nigeria. The majority of ovarian tumours in young girls here are lymphosarcomas. The same is not true of adult women.

We are grateful to the medical staff of the Department of Obstetrics and Gynaecology and to other surgeons and physicians for permission to use their clinical records, to Dr. W. P. Cockshott for radiological reports and to Mr. F. E. Speed of the Medical Illustration Unit, University College Hospital, Ibadan, for the photographs.

### REFERENCES

Burkitt, D.—(1958) *Brit. J. Surg.*, **197**, 218.

Camain, R.—(1954) *Bull. Soc. Pat. exot.*, **47**, 614.

Davies, J. N. P.—(1959) in ' Modern Trends in Pathology ', ed. D. H. Collins, London (Butterworth & Co.), p. 153.

*Idem* and Wilson, Barbara A.—(1954) *E. Afr. med. J.*, **31**, 395.

*Idem*, Wilson, B. A. and Knowelden, J.—(1958) *Brit. med. J.*, **2**, 439.

Edington, G. M.—(1956) *Brit. J. Cancer*, **10**, 595.

Elmes, B. G. T., and Baldwin, R. B. T.—(1947) *Ann. trop. Med. Parasit.*, **41**, 321.

Haines, M.—(1958) in ' Cancer ', ed. R. W. Raven. London (Butterworth & Co.), Vol. 2, p. 298.

Harnett, W. L.—(1952) ' A Survey of Cancer in London '. London (British Empire Cancer Campaign).

Higginson, J. and Oettlé, A. G.—(1960) *J. nat. Cancer. Inst.*, **24**, 589.

Nelson, G. A., Dockerty, M. B., Pratt, J. H. and ReMine, W. H.—(1958) *Amer. J. Obstet., Gynec.*

O'Conor, G. T. and Davies, J. N. P.—(1960) *J. Pediat.*, **56**, 526.

Rosenberg, S. A., Diamond, H. D., Dargeon, H. W. and Craver, L. F.—(1958) *New Engl. J. Med.*, **295**, 505.

Steiner, P. E.—(1954) ' Cancer : Race and Geography '. Baltimore (Williams & Wilkins Co.).

Symmers, W. St C.—(1958) in ' Cancer ', ed. R. W. Raven. London (Butterworth & Co.), Vol. 2, p. 448.